# Parental Attitudes and Digital Parenting in the Early Years: Development and Validation of the PADTS Scale

**DOI:** 10.1111/cch.70199

**Published:** 2026-01-21

**Authors:** Katrina McLaughlin, Lisa Bunting, Paul Connolly, Karen Winter, Rosie Flewitt, Sandra El Gemayel, Lorna Arnott, Andrea Dalziell, Julia Gillen, Janet Goodall, Min‐Chen Liu, Sabina Savadova, Sarah Timmins

**Affiliations:** ^1^ School of Psychology Queen's University Belfast Belfast UK; ^2^ School of Social Sciences, Education and Social Work Queen's University Belfast Belfast UK; ^3^ Faculty of Arts, Humanities and Social Sciences Ulster University Belfast UK; ^4^ Education and Social Research Institute (ESRI), Birley Fields Campus Manchester Metropolitan University Manchester UK; ^5^ Education and Social Research Institute Manchester Metropolitan University Manchester UK; ^6^ Institute of Education University of Strathclyde Glasgow UK; ^7^ School of Psychological Sciences and Health University of Strathclyde Glasgow Scotland UK; ^8^ Department of Linguistics and English Language Lancaster University Lancaster UK; ^9^ School of Social Sciences Swansea University Swansea UK; ^10^ Childhood Studies and Practice, Holyrood Road Moray House School of Education and Sport Edinburgh UK; ^11^ Department for Education and Childhood Studies Swansea University Swansea UK

**Keywords:** digital parenting, early years education, parental attitudes, psychometric scale development, young childrens technology use

## Abstract

**Background:**

This paper reports on the development and validation of the 15‐item Parental Attitudes to Digital Technology Scale (PADTS), a brief, psychometrically validated measure assessing parents' beliefs confidence, and concerns about their very young children's use of digital technologies.

**Method:**

Developed as part of the UK‐wide Toddlers, Tech and Talk (TTT) study, PADTS addresses a gap in existing research by focusing on children from birth to 3 years, a stage often overlooked in digital parenting literature. Co‐developed with parents and early years experts, the scale was tested with a nationally balanced UK sample (*N* = 934).

**Results:**

Exploratory and confirmatory factor analyses supported a four‐factor structure: perceived risks, perceived learning benefits, parental confidence and technology‐related anxiety. The PADTS showed strong model fit and measurement invariance across parent gender, ethnicity and region, with some variation by child age. Correlational analyses indicated that benefits, perceptions and confidence were associated with supportive digital parenting, while anxiety was more weakly linked.

**Conclusion:**

PADTS shows potential as a practical tool for researchers, practitioners and policy‐makers and may support a more nuanced understanding of how parental attitudes shape early digital experiences.

## Summary

- This paper introduces the Parental Attitudes to Digital Technology Scale (PADTS), a concise, conceptually grounded and psychometrically validated tool designed for parents of children aged 0–3 years.
- The study identified four distinct factors underpinning parental attitudes: perceived risks of digital technology, perceived learning benefits, parental digital confidence, and technology‐related anxiety.
- The study demonstrated strong model fit, internal reliability and measurement invariance across parent gender, ethnicity and UK region, with some age‐related variation highlighting the developmental relevance of parental beliefs.
- The PADTS can assist early years practitioners, health visitors and family support workers in identifying and addressing both concerns and strengths in parents' digital attitudes.
- The scale offers a reliable means of identifying parental support needs, informing the design of tailored, strengths‐based resources and messaging that reflect diverse beliefs and family contexts.

## 1 Introduction

### 1.1 Importance of Parental Attitudes Toward Digital Technology

Parental attitudes are central to shaping children's access to and use of digital technologies in the home. Positive parental attitudes are linked to higher ownership and use of digital devices by children, while negative attitudes often result in children's limited access or restricted use (Konca 2022; Akgün 2023). Parental beliefs also shape engagement, where parents who value digital literacy tend to support more active and purposeful use of digital technologies (Dong et al. 2022; Lauricella et al. 2020). Digital engagement bolsters children's confidence and skills and encourages the development of safe and independent digital practices (Kumpulainen et al. 2020; Livingstone et al. 2015). Digital engagement in the early years is associated with key developmental outcomes including emergent literacy, creativity and communicative competence, particularly when parents actively support such use (Papadakis et al. 2019).

### 1.2 Factors Influencing Parental Attitudes

Parental attitudes toward digital technology are shaped by a complex set of factors (Johnson and Puplampu 2008). Drawing upon Bronfenbrenner's (Bronfenbrenner 1994) bioecological systems theory, Johnson and Puplampu (2008) propose the ‘ecological techno‐subsystem’, which places digital media exposure within the child's microsystem. This framework, further refined by Johnson (2010) into the ecological techno‐microsystem, encompasses the interplay between digital media, family contexts and children's developmental trajectories.

At the microsystem level, studies illustrate the role played by family dynamics and child temperament. For example, Shin et al. (2021) found that toddlers' screen use was linked to maternal stress, with stress mediating the relationship between child temperament and screen time. Importantly, the nature of digital use, rather than the technology itself, shapes parental attitudes. O'Connor and Fotakopoulou (2016) found that parents were more accepting of devices when used to aid communication with family or for specific tasks such as taking photographs. These findings suggest that parental attitudes toward technology appear somewhat nuanced; often hinging on perceived functionality and context of use, rather than blanket approval or disapproval.

At the macrosystem level, cultural and national differences are shown to influence attitudes (Mallawaarachchi et al. 2022). These attitudes often vary between countries and are guided by broader educational values and policy contexts (Dardanou et al. 2020). In the Philippines, Dy et al. (2023) showed that while parents sometimes blamed devices for their children's challenging behaviours, they did not necessarily limit their screen time exposure. Parental demographics such as parental education, age and income have also been revealed to correlate with both attitudes and patterns of child screen use (Wiltshire et al. 2021; Zakaria et al. 2022). For example, Wiltshire et al. (2021) found that lower levels of parental education were associated with earlier infant screen exposure and a greater reliance on screens to manage daily routines. A recent RCT in India showed that early intervention in the form of parental education can help limit screen time in the first 2 years (Poonia et al. 2024). However, Mekhail et al. (2024) revealed that despite parents having concerns about their children's screen time, digital devices are very much embedded in daily routines, seen as the social norm, and thus are difficult to remove. Thus, as Flewitt and Clark (2020) argue, very young children's home learning environments are digitally networked spaces with multiple external influences.

### 1.3 Ambivalence and Anxiety in Parental Attitudes

Many studies reveal a tension within the realm of parental attitudes, ranging from approval and valuing technology's potential to feeling anxious and fearing its risks. Murphy and Headley (2020) found that while parents of toddlers often saw benefits to tech (e.g., coordination and communication), they were simultaneously anxious about addiction, cost and health. Available guidelines highlight a risk‐averse approach toward digital technology. For example, the World Health Organization guidelines (WHO 2019) state that for 1‐year‐old children, sedentary screen time (such as watching TV or videos, playing computer games) is not recommended. For those aged 2 years, sedentary screen time should be no more than 1 hour and that less is better. In a UK context, the recently published DfE guidelines (DfE 2025) incorporate the WHO Guidelines outlined above. However, as Heller (2021) points out, the majority of parents do not follow these guidelines, and many are not even aware that they exist. O'Connor and Fotakopoulou (2016) found that most parents reported lacking formal advice on safe screen time for toddlers, instead relying on instinct or conflicting input from peers and professionals. Palaiologou (2016) similarly highlighted how contradictory messages from educators left parents feeling uncertain and unsupported in their decisions. These mixed messages may result in parents overly restricting or avoiding digital devices entirely.

### 1.4 The Role of Parental Confidence

Parental self‐efficacy plays a key role in shaping both attitudes and mediation strategies. Nicholas and Paatsch (2018) found that parents were far more confident using printed texts with their children than electronic formats, despite being operationally competent with tech. Neumann et al. (2020) argue that many parents feel confident in device operation but are unsure how to select high‐quality content or support balanced screen time. This gap between operational skills and educational competence can act as a barrier to effective digital engagement in the home.

Parental confidence also influences the consistency and quality of mediation. Parents who feel equipped to support digital activities are more likely to engage in co‐use, suggest educational apps or explain screen content—practices shown to benefit children's cognitive and language development (Kumpulainen et al. 2020; Papadakis et al. 2019). In contrast, low confidence may result in inconsistent limits, reliance on devices as a distraction or missed opportunities to support digital play as a learning resource. This is particularly salient for parents of children with additional needs. Apps et al. (2024) found that these parents often saw digital technology as beneficial for communication, regulation or accessibility but also reported heightened concerns about online safety and content quality. These dual perspectives highlight the importance of tools that can capture the complex and multifaceted nature of parental attitudes.

### 1.5 Existing Measures of Parental Attitudes

A number of scales have been developed to assess parental attitudes and behaviours related to digital parenting, with varying constructs, populations and focus. The Digital Parenting Attitude Scale (DPSAS), widely used in Turkish studies, includes constructs on promoting effective digital media use and protecting children from risks (Fidan and Olur 2023; Altindağ Kumaş and Sardohan Yildirim 2024). Other established tools include the Parents' Attitudes Toward Children's Use of ICT Scale (PACU‐ICT) (Gür and Türel 2022), the Media and Technology Usage and Attitudes Scale (Rosen et al. 2013) and the Digital Parenting Awareness Scale (Manap and Durmuş 2020). However, many of these instruments are designed for older children, lack age specificity or conflate general technology use with parenting concerns.

Some more recent efforts have addressed these limitations (Navarro et al. 2023; Bulduk et al. 2025). For example, Bulduk et al. (2025) developed a Parental Knowledge‐Attitude Scale specifically for parents of children aged 6 months to 6 years. However, this was not yet available during the design phase of PADTS. Moreover, most existing tools do not disaggregate attitudes into distinct cognitive, affective and self‐efficacy domains, limiting their explanatory power in intervention and applied contexts.

The PADTS was designed to address these gaps by capturing three core dimensions—Perceived Risks of Digital Technology, Parental Digital Confidence and Technology‐Related Anxiety, and by focusing specifically on parents of children aged from birth to 3 years. In doing so, it offers a theoretically grounded and practically useful tool for understanding how parental dispositions shape early digital experiences and, by extension, early educational opportunities.

## 2 Present Study

Based on the literature, there remains a clear need for a psychometrically sound, conceptually clear and age‐appropriate measure of parental attitudes toward digital technology specifically designed for early childhood. Firstly, most existing tools are developed for parents of older children or take a general approach to digital parenting, without addressing the unique concerns and uncertainties experienced by parents of children from birth to 3 years (e.g., Neumann et al. 2020; Livingstone and Zhang 2021). Secondly, many scales do not distinguish between different psychological dimensions, such as cognitive beliefs, affective concerns and parental self‐efficacy—which are crucial for understanding and supporting digital parenting in applied contexts (Nicholas and Paatsch 2018; Apps et al. 2024). Thirdly, only a small number of recent scales (e.g., Bulduk et al. 2025; Navarro et al. 2023) target the early years, and most of these were unavailable at the time of this study's design. Moreover, some rely on single‐factor models or lack clarity in item phrasing and factor structure.

The PADTS was designed to address this gap by capturing four core dimensions, perceived risks of digital technology, perceived learning benefits, parental digital confidence and technology‐related anxiety, and by focusing specifically on parents of children aged from birth to 3 years. In doing so, it offers a theoretically grounded and practically useful tool for understanding how parental dispositions shape early digital experiences and, by extension, early educational opportunities.

While the primary aim of this study was scale development and validation, two theoretically informed expectations guided our validation analyses. First, it was anticipated that parental digital confidence would correlate more strongly with supportive digital parenting behaviours (e.g., co‐play and scaffolding) than with technology‐related anxiety, consistent with previous evidence on the predictive power of self‐efficacy beliefs (Bandura 1977). Second, it was expected that perceived learning benefits would be more salient among parents of older children, reflecting their greater exposure to direct child–technology interaction.

## 3 Development of the Scale, Subscales and Items

Scale development is a rigorous and developmental process (Badenes‐Ribera et al. 2020). A literature review was conducted to determine the need for a new scale, what measures exist already and if the proposed measure was conceptually distinct (Zickar 2020). The next step was to define the construct/s being measured, which then guided the item writing. In line with best practice, items were kept clear and simple (Haladyna and Rodriguez 2013).

The draft items were presented to an interdisciplinary team of experts in both the target constructs and psychometric measure development. Items were reviewed for clarity, coherence and conceptual relevance (Pérez‐Rivas et al. 2023), with ambiguous, redundant or misaligned items removed or revised. The refined item set was piloted with a diverse group of parents of children aged 0–3 years (72 partial responses, 45 complete responses) recruited through convenience sampling across the United Kingdom. In the pilot, three conceptual domains were tested separately using principal component analysis (PCA): attitudes/well‐being (including both risk‐ and benefit‐framed items), parental confidence and parental anxiety. Each domain showed a single clear factor with strong loadings (> 0.73), explaining between 62% and 68% of the variance. Sampling adequacy was high across all scales (KMO = 0.767–0.844; Bartlett's *p* < 0.001). Expert review of the pilot results recommended two refinements: separating risk‐framed and benefit‐framed items in the attitudes/well‐being scale to create distinct attitudes/well‐being and learning/benefits subscales and retaining confidence and anxiety as separate subscales to reflect their conceptual distinction.

A subsequent PCA of the four‐factor structure confirmed strong internal coherence and sampling adequacy (KMO = 0.767–0.844; Bartlett's χ^2^(190) = 3437, *p* < 0.001). All four factors exceeded the Kaiser criterion with eigenvalues > 1, and each explained between 62% and 68% of variance. Factor loadings were uniformly strong (lowest loading = 0.67), with no problematic cross‐loadings. Qualitative feedback from 10 parents confirmed that the questionnaire was accessible, clear and comprehensive, supporting its use in the main survey (Table 1). All items were scored on a 5‐point Likert scale from *strongly disagree* (1) to *strongly agree* (5). After reverse coding as appropriate, higher scores reflect more positive attitudes toward digital technology and child well‐being and learning, greater confidence in supporting children's digital engagement and higher levels of technology‐related anxiety.

**TABLE 1:** Parental attitudes to digital technology scale (PADTS)—Original.

**Health and well‐being subscale**
1. Digital devices are damaging to children's mental health
2. Young children use digital technology too much, too early
3 Digital devices offer opportunities for young children to have fun^a^
4. Digital devices are damaging to children's physical health
5. Digital devices are not damaging to children's social and emotional development^a^
**Learning subscale**
1. Digital devices offer opportunities for young children to develop skills with numbers
2. Digital devices are damaging to children's learning
3. Digital devices offer opportunities for young children to develop skills with reading^a^
4. Digital devices are not suitable for young children to use
5. Digital devices offer opportunities for young children to develop creative skills (e.g., drawing, painting, taking photos, making short videos etc.)^a^
**Parent confidence subscale**
1. I do not have enough information about how to keep my child safe when using digital technology
2. I know where I can access support and advice around children's digital usage^a^
3. I do not feel competent in teaching my child how to use digital devices
4. I know how to keep my child safe when using digital technology^a^
5. I believe I have all the skills to support my child using digital devices^a^
**Parent anxiety subscale**
1. I get anxious when my child is spending too long on digital devices^a^
2. I do not have any worries about my child using digital devices as there are no risks with this age
3. I am concerned that excessive use of digital devices will negatively impact the amount of time my child spends socialising with other children and adults^a^
4. I do not worry about my child's use of digital devices at this age
5. I worry about the inappropriate content that my child might access online^a^

^a^ Reverse scored.

## 4 Factor Structure and Scale Validation

The Parental Attitudes to Digital Technology Scale (PADTS) was validated using data from the Toddlers, Tech and Talk study (Flewitt et al. 2024) Phase 1 survey (for details of survey participant recruitment, administration and completion; see Winter et al. 2025). In total, 1444 valid responses were provided to the survey, and all subsequent analyses were conducted in SPSS V29 and Jamovi V2.4.11.

The survey data (*N* = 1444) comprised both a panel survey (*n* = 934) and an open online survey (*n* = 510). Following an initial exploratory factor analysis (EFA) conducted on the full sample, subsequent reliability and validity testing focused on the panel sample, which was recruited using quota sampling to ensure equal representation across the four UK nations (England, Scotland, Wales and Northern Ireland). Within the panel sample, 49.8% of children were reported as female, and 16.1% were from minority ethnic backgrounds. While the sample was well balanced geographically (26.5% England, 24.5% Scotland, 24.7% Wales and 24.3% Northern Ireland), the age distribution was less even despite our best efforts: 51.0% of children were aged 2–3 years, compared to 28.6% aged 1–2 years and 20.4% under 1 year (*M* = 2.31, SD = 0.79, range = 2). This age skew should be considered when interpreting results by developmental stage.

### 4.1 Exploratory Factor Analysis

A split‐half approach was adopted, with exploratory factor analysis conducted on one‐half of the panel sample (*n* = 466). As it was anticipated that the factors (e.g., risk, confidence and anxiety) would be correlated, this was conducted using Maximum Likelihood extraction with oblimin rotation. Bartlett's test of sphericity was significant [*χ* ^2^(190, *N* = 466) = 3437, *p* < 0.001], and KMO values ranged from 0.751 to 0.890, confirming sampling adequacy.

During model refinement, five negatively worded items using ‘I do not …’ constructions were removed due to semantic ambiguity and potential method effects (see Tables S5–S7 for factor loadings and fit statistics). These included two items from the confidence subscale, two from anxiety and one from the well‐being domain. Although reverse‐coded items are traditionally used to reduce acquiescence bias, evidence suggests they can introduce confusion and inflate method variance, particularly when surrounded by positively worded items (Suárez Álvarez et al. 2018).

Importantly, this decision also reflected a theoretical distinction: that positive and negative attitudes toward children's digital device use are not opposites but distinct constructs. For instance, a parent might simultaneously worry about screen time while also recognising the learning potential of digital apps. This multidimensional perspective aligns with contemporary digital parenting literature and the final four‐factor model retained a balanced structure, with each domain comprising three to five items.

The resulting four‐factor model (Table 2) comprised the following:

- Well‐being concerns (five items)
- Perceived learning benefits (four items)
- Parental digital confidence (three items)
- Digital technology‐related anxiety (three items)

**TABLE 2:** Parental attitudes to digital technology scale (PADTS)—Validated structure.

**Well‐being concerns (five items)**
1. Digital devices are damaging to children's mental health
2. Young children use digital technology too much, too early
3. Digital devices are damaging to children's physical health
4. Digital devices are damaging to children's learning
5. Digital devices are not suitable for young children to use
**Perceived learning benefits (four items)**
1. Digital devices offer opportunities for young children to have fun^a^
2. Digital devices offer opportunities for young children to develop skills with numbers^a^
3. Digital devices offer opportunities for young children to develop skills with reading^a^
4. Digital devices offer opportunities for young children to develop creative skills (e.g., drawing, painting, taking photos, making short videos etc.)^a^
**Parental digital confidence (three items)**
1. I know where I can access support and advice around children's digital usage^a^
2. I know how to keep my child safe when using digital technology^a^
3. I believe I have all the skills to support my child using digital devices^a^
**Digital technology‐related anxiety (three items)**
1. I get anxious when my child is spending too long on digital devices.
2. I am concerned that excessive use of digital devices will negatively impact the amount of time my child spends socialising with other children and adults.
3. I worry about the inappropriate content that my child might access online.

^a^ Reverse scored.

Although one item, ‘Digital devices are damaging to children's learning’, references learning directly, it was retained within the well‐being concerns factor for both empirical and theoretical reasons. Conceptually, the phrasing emphasises harm and risk, framing learning in a deficit‐focused way that aligns more closely with the tone and content of the other well‐being‐oriented items (e.g., mental health and physical harm).

### 4.2 Confirmatory Factor Analysis

CFA confirmed the four‐factor structure in both split and full samples (e.g., full sample: CFI = 0.948, RMSEA = 0.057; see Tables S8 and S9).

Model comparisons demonstrated that the revised 15‐item four‐factor model substantially outperformed both the original 20‐item model and a single‐factor baseline model. All factor loadings were significant and ranged from 0.465 to 0.913. Internal consistency was good across scales (*α* = 0.76–0.85), with anxiety acceptable (*α* = 0.65) given its brevity.

### 4.3 Associations With Parenting Behaviours and Device Use

Correlations between the four PADTS subscales and parenting behaviours (Tables 3 and 4) revealed expected and yet nuanced patterns.

**TABLE 3:** Correlation matrix of relationships between attitudes, device ownership and usage (*N* = 934).

	1)	2)	3)	4)	5)	6)	7)	8)	9)
1)	1								
2)	0.373**	1							
3)	0.361**	0.355**	1						
4)	0.203**	0.360**	0.282**	1					
5)	0.212**	0.383**	0.339**	0.555**	1				
6)	0.047	−0.067*	0.199**	0.067*	0.068**	1			
7)	0.075**	0.170**	0.190**	0.193**	0.203**	0.387**	1		
8)	0.037	0.123**	0.069**	0.098**	0.092**	0.115**	0.282**	1	
9)	0.001	−0.023	0.094**	0.044	0.049	0.502**	0.129**	0.162**	1

*Note:* 1) Range of devices in home. 2) Range of devices child own. 3) Where child uses devices. 4) Often plays with child. 5) Child often plays alone. 6) Risks. 7) Benefits. 8) Confidence. 9) Anxiety.

**TABLE 4:** Correlation matrix of relationships between attitudes toward risk, benefits, confidence, anxiety and parent digital support (*N* = 934).

	1	2	3	4	5	6	7	8	9	10	11	12	13	14	15	16	17	18	19	20	21
1	1																				
2	0.387**	1																			
3	0.115**	0.282**	1																		
4	0.502**	0.129**	0.162**	1																	
5	0.131**	0.262**	0.146**	0.041	1																
6	0.145**	0.210**	0.104**	0.056*	0.364**	1															
7	0.060*	0.085**	−0.023	−0.007	0.037	0.089**	1														
8	0.228**	0.228**	0.097**	0.066*	0.260**	0.224**	0.265**	1													
9	0.093**	0.117**	0.023	−0.017	0.176**	0.212**	0.182**	0.279**	1												
10	0.161**	0.174**	0.069**	0.071**	0.252**	0.247**	0.132**	0.257**	0.177**	1											
11	0.167**	0.195**	0.112**	0.012	0.444**	0.302**	0.100**	0.270**	0.247**	0.260**	1										
12	0.159**	0.143**	0.078**	−0.011	0.126**	0.140**	0.162**	0.265**	0.325**	0.169**	0.244**	1									
13	0.203**	0.195**	0.135**	0.066*	0.251**	0.255**	0.149**	0.266**	0.288**	0.214**	0.324**	0.413**	1								
14	0.169**	0.198**	0.137**	0.012	0.409**	0.380**	0.121**	0.304**	0.295**	0.337**	0.474**	0.303**	0.409**	1							
15	0.174**	0.161**	0.064*	0.002	0.165**	0.103**	0.023	0.185**	0.181**	0.288**	0.215**	0.202**	0.177**	0.235**	1						
16	0.144**	0.163**	0.110**	0.042	0.322**	0.300**	0.110**	0.235**	0.195**	0.282**	0.407**	0.217**	0.285**	0.411**	0.123**	1					
17	0.250**	0.233**	0.122**	0.082**	0.334**	0.347**	0.132**	0.349**	0.257**	0.354**	0.401**	0.316**	0.415**	0.458**	0.259**	0.404**	1				
18	0.131**	0.198**	0.152**	−0.008	0.419**	0.333**	0.110**	0.250**	0.235**	0.266**	0.549**	0.235**	0.318**	0.481**	0.171**	0.443**	0.397**	1			
19	0.114**	0.108**	0.037	−0.049	0.142**	0.135**	0.166**	0.232**	0.260**	0.178**	0.218**	0.319**	0.317**	0.278**	0.183**	0.231**	0.358**	0.249**	1		
20	0.100**	0.107**	0.076**	−0.04	0.217**	0.212**	0.125**	0.255**	0.265**	0.215**	0.311**	0.260**	0.321**	0.345**	0.260**	0.261**	0.350**	0.324**	0.480**	1	
21	0.164**	0.154**	0.133**	0.026	0.248**	0.187**	0.045	0.214**	0.141**	0.217**	0.321**	0.260**	0.319**	0.335**	0.259**	0.279**	0.378**	0.331**	0.344**	0.442**	1

*Note:* 1) Risks. 2) Benefits. 3) Confidence. 4) Anxiety. 5) Suggest fun games, apps or activities which my child will enjoy. 6) Join my child in the games or activities they want to do. 7) Help my child physically to hold or move the device. 8) Show my child how to use the device (e.g., touch, tap and slide). 9) Talk with my child about the content or about what they are doing 10) Sit with my child without interfering unless they get stuck. 11) Suggest games, apps or activities which I think will help my child to learn. 12) Point to items on the screen and name or explain them to my child. 13) Help my child to learn words, letters, sounds, shapes and colours. 14) Help my child to solve problems in their game or activity. 15) Leave my child to use the device on their own so I can get on with something else (e.g., wash up, make a phone call, attend to a sibling etc.). 16) Encourage my child to complete a task. 17) Praise my child when they do something well. 18) Suggest games, apps or activities that encourage my child to be imaginative or creative. 19) Supervise my child's device use (e.g., stay in the room, keep an eye on what child is doing). 20) Set limits on my child's use (e.g., how long they can play, which apps they can use). 21) Set parent controls in the device my child uses to make sure my child is safe. *p* < 0.05 is indicated by *. *p* < 0.05 is indicated by **.

Risks and anxiety were strongly correlated, but risk perceptions also correlated positively with confidence and benefits, suggesting ambivalence is common. Confidence was moderately related to benefits but only weakly to anxiety, reinforcing its independence from worry.

In relation to device ownership and usage, parents who perceived more benefits and felt more confident were more likely to co‐play with their child and have broader home access and usage contexts. Risk perceptions also showed modest positive associations, while anxiety was only weakly related to usage or co‐play, suggesting that worry does not predict behaviour directly.

In terms of parental support behaviours, we have the following:

- Risks and benefits were both positively associated with scaffolding behaviours such as praising, suggesting games and helping solve problems.
- Confidence showed smaller but consistent associations with active support (e.g., encouraging, demonstrating, praising).
- Anxiety again showed weak and inconsistent links with support, reinforcing its limited behavioural influence in this context.
- Together, these findings suggest that risks, benefits and confidence are particularly relevant for predicting parental involvement, while anxiety may function more as an affective response than a behavioural driver.

### 4.4 Measurement Invariance Testing

Multigroup CFA assessed the PADTS structure across parent gender (Male/Female), ethnicity (White/BME), UK nation (England/Scotland/Wales/Northern Ireland) and child age group (0–1 year, 1–2 years, 2–3 years). The four‐factor model demonstrated likely invariance across gender, ethnicity and region (ΔCFI < 0.01; see Table S11). However, metric noninvariance was observed across child age, with changes in CFI exceeding the accepted threshold (ΔCFI = 0.023).

Examination of the measurement model revealed that items reflecting learning‐related harms and benefits varied in strength across child age groups. The metric noninvariance observed across child age groups is theoretically consistent with known developmental and parenting differences during the early years. Parents of infants are likely to approach digital technology with more generalised concerns and uncertainties, whereas parents of toddlers may have more direct experience with their child's engagement and thus form more differentiated attitudes, particularly around learning benefits and potential harms. These shifts in perspective likely account for differences in factor loadings across age groups. Notably, the sample was skewed toward older children, with 51.0% aged 2–3 years, which may have further influenced the factor structure and inflated the salience of developmentally advanced attitudes. While this limits strict comparability of scores between age groups, it also likely reflects meaningful variation in how parental attitudes evolve as children grow and gain independence.

### 4.5 Alternative Model Testing

Given that the pilot PCA had originally been organised around three conceptual domains, an alternative three‐factor model was also specified for validation, enabling direct comparison of model fit, conceptual coverage and measurement invariance. This model excluded several items due to semantic ambiguity and concerns over potential method artefacts associated with reverse‐coded items in the attitudes to well‐being and learning domains. In the resulting nine‐item model, the learning/benefits items were removed during item reduction, producing a structure comprising: (1) attitudes/well‐being (primarily risk‐framed attitudes toward digital devices) (2) parental confidence and (3) technology‐related anxiety. While this model achieved superior fit indices (CFI = 0.978, RMSEA = 0.51; see Tables S12–S16 for factor loadings, fit statistics and invariance testing and Table S17 for CFA model comparisons) and demonstrated greater measurement invariance across child age, the omission of the learning/benefits construct limited its conceptual coverage. On this basis, the four‐factor model was retained as the preferred structure. Nonetheless, the three‐factor version may offer a useful alternative for future research that prioritises brevity, stronger invariance properties or large‐scale population screening.

## 5 Discussion

This study developed and validated the PADTS, a brief, conceptually grounded tool to assess how parents of children aged from birth to 3 years perceive and manage their children's digital technology use. Using a UK‐balanced panel sample, exploratory and confirmatory analyses supported a four‐factor structure: perceived risks of digital technology, perceived learning benefits, parental digital confidence and technology‐related anxiety.

EFA highlighted that negatively phrased, reverse‐coded items introduced response artefacts, with such items clustering independently of the conceptual domain. Five items using complex ‘I do not …’ constructions were removed based on poor fit and a theoretical decision to treat positive and negative attitudes as co‐existing, not opposite. The refined 15‐item scale showed strong internal consistency across most subscales (*α* = 0.76–0.85), with acceptable reliability for the shorter anxiety subscale (*α* = 0.65).

CFA confirmed the four‐factor model (CFI = 0.948, RMSEA = 0.057) and showed it outperformed both the original 20‐item version and a one‐factor baseline, providing a more interpretable and efficient measure. Correlations between PADTS scales revealed expected patterns: risks and anxiety were strongly associated, while confidence and benefits also correlated positively. These findings reflect the ambivalence often seen in digital parenting; parents may simultaneously hold concerns and perceive value.

Confidence and benefit perceptions were more predictive of parental behaviour, correlating with co‐play, access to devices and usage contexts, while risk attitudes showed modest associations. Support behaviours, including scaffolding and co‐engagement were associated with confidence and both risk and benefit beliefs. Consistent with our hypothesis, confidence was more strongly associated with supportive digital parenting behaviours than anxiety. Importantly, and in line with our second expectation, anxiety showed no meaningful associations with actual parenting behaviours such as co‐play or support strategies, highlighting a theoretically significant distinction between emotional concern and parental action. This suggests that while anxiety may reflect underlying worry or uncertainty, it does not translate directly into observable parenting practices, indicating the need for a more nuanced approach to, and measurement of parental attitudes.

Multigroup CFA demonstrated invariance across gender, ethnicity and UK region. However, metric noninvariance emerged across child age, particularly for learning‐related items. These items loaded more strongly for parents of older children in the birth to 3 years age range, suggesting that attitudes may become more differentiated as children grow. This finding should be interpreted in the context of a skewed age distribution, with over half (51%) of the sample comprising children aged 2–3 years, which may have amplified the developmental salience of certain items. Although this limits the direct comparability of scores across age groups, it likely captures meaningful shifts in parental attitudes as children develop and engage more independently with digital devices. As such, the PADTS may be most appropriately used to examine patterns within age groups or track changes in attitudes over time, rather than to directly compare parents of children at different developmental stages.

## 6 Implications for Research and Practice

The PADTS provides a valuable tool for researchers and early years practitioners (including nursery staff, preschool teachers, childminders, early years teachers and teaching assistants) aiming to understand and support digital engagement at home. It captures distinct domains, risks, benefits, confidence and anxiety that shape parenting strategies and media mediation.

The scale can be used to assess the impact of interventions (e.g., parenting workshops, school‐home initiatives) and inform the design of digital resources. Its multidimensional structure offers greater insight than existing tools such as the PACU‐ICT (Fidan and Olur 2023) or the Parental Knowledge‐Attitude Scale (Bulduk et al. 2025), particularly by separating cognitive, affective and self‐efficacy components. Robust psychometric validation and invariance testing strengthen its suitability for applied and research contexts.

In practical settings, PADTS may be used for brief screening in health visitor appointments, early childhood settings or family support services to identify parents who are highly anxious, overly risk‐focused or lacking confidence in digital parenting. It could also inform tailored guidance or discussion points in antenatal or postnatal groups, as well as targeted messaging or resources to promote balanced, developmentally appropriate digital use in the home. The scale could support reflective practice among early years professionals or be embedded in digital literacy components of teacher training to improve understanding of parental perspectives.

## 7 Limitations and Future Directions

While the PADTS shows strong psychometric performance, the anxiety subscale may benefit from future item refinement. Self‐report data and the cross‐sectional design also limit inference; longitudinal or observational validation would strengthen future applications of the measure. In addition, the sensitivity of the topic may introduce social desirability bias, whereby parents respond in line with perceived norms or expectations rather than reflecting their actual beliefs or practices. This should be considered when interpreting scores, particularly for items related to screen time, risks and parental anxiety.

A shorter three‐factor version showed slightly stronger fit and invariance but excluded learning benefits, a conceptually and empirically distinct domain. The four‐factor model was therefore retained. However, the three‐factor version may prove useful for rapid screening or large‐scale use. Future research should test the PADTS in cross‐cultural contexts, examine its predictive value for child outcomes and explore how parental attitudes shift in response to policy and/or technological change.

## 8 Conclusion

The PADTS provides a concise, psychometrically robust measure of how parents of children from birth to age 3 years perceive and approach digital technology use. Its four‐factor structure—covering risks, benefits, confidence and anxiety—captures core psychological orientations relevant to digital mediation, parenting support and early learning environments. While further longitudinal validation is needed, the PADTS has the potential to provide a sound foundation for both academic research and applied practice in the early years digital landscape. Future work should explore cultural adaptation and validation of the PADTS across international contexts to ensure its relevance and utility in diverse family and policy environments.

## Author Contributions

**Katrina McLaughlin:** conceptualization, funding acquisition, methodology, writing – original draft, formal analysis, writing – review and editing. **Lisa Bunting:** conceptualization, writing – original draft, methodology, formal analysis, writing – review and editing. **Paul Connolly:** conceptualization, methodology, formal analysis, writing – review and editing. **Karen Winter:** conceptualization, funding acquisition, methodology, writing – original draft, writing – review and editing. **Rosie Flewitt:** funding acquisition, methodology, writing – review and editing. **Sandra El Gemayel:** methodology, writing – review and editing. **Lorna Arnott:** funding acquisition, methodology, writing – review and editing. **Andrea Dalziell:** writing – review and editing. **Julia Gillen:** funding acquisition, methodology, writing – review and editing. **Janet Goodall:** funding acquisition, methodology, writing – review and editing. **Min‐Chen Liu:** formal analysis, writing – review and editing. **Sabina Savadova:** writing – review and editing. **Sarah Timmins:** writing – review and editing.

## Funding

This work was supported by the Economic and Social Research Council (grant number ES/W001020/1).

## Ethics Statement

All aspects of the project have been approved by the ethics committees of all partner institutions involved.

## Conflicts of Interest

The authors declare no conflicts of interest.

## AI Use Statement

AI tools (ChatGPT by OpenAI) were used to support the editing and refinement of written content, including rephrasing for clarity, summarising reviewer‐style feedback and checking reference consistency. All analytical decisions, conceptual development, interpretation of findings and final wording were made by the authors. The AI was not used to generate original research content, data analysis, or conclusions.

## Supporting information


**Table S5:** Split sample factor loadings for exploratory factor analysis with 20 items (*N* = 466).
**Table S6:** Split sample factor loadings for exploratory factor analysis with 15 items (N = 466).
**Table S7:** Model statistics for EFA models (N = 466).
**Table S8:** Split sample factor loadings for CFA with 15 items (N = 466).
**Table S9:** Full sample factor loadings for CFA with 15 items (N = 932).
**Table S10:** Model statistics for CFA models (N = 932).
**Table S11:** Fit statistics for unconstrained and constrained model.
**Table S12:** Split sample factor loadings for exploratory factor analysis with nine items (N = 466).
**Table S13:** Model statistics for EFA models (N = 466).
**Table S14:** Model statistics for EFA models (N = 466).
**Table S15:** Split sample factor loadings for CFA with nine items (N = 466).
**Table S16:** Full sample factor loadings for CFA with nine items (N = 934).
**Table S17:** Model statistics for EFA models (N = 466).
**Table S18:** Fit statistics for unconstrained and constrained model.

## Data Availability

The data are available on request and have been deposited on the UK Data Service in line with funding requirements from the Economic and Social Research Council.
