# Supplementary material for: Parental Attitudes and Digital Parenting in the Early Years: Development and Validation of the PADTS Scale
Source: Child Care Health Dev. 2026 Jan 21;52(1):e70199. doi: 10.1111/cch.70199 (PMC12821080; doi:10.1111/cch.70199)
Supplement: Supplementary file 1 — Table S5: Split sample factor loadings for exploratory factor analysis with 20 items (N = 466). Table S6: Split sample factor loadings for exploratory factor analysis with 15 items (N = 466). Table S7: Model statistics for EFA models (N = 466). Table S8: Split sample factor loadings for CFA with 15 items (N = 466). Table S9: Full sample factor loadings for CFA with 15 items (N = 932). Table S10: Model statistics for CFA models (N = 932). Table S11: Fit statistics for unconstrained and constrained model. Table S12: Split sample factor loadings for exploratory factor analysis with nine items (N = 466). Table S13: Model statistics for EFA models (N = 466). Table S14: Model statistics for EFA models (N = 466). Table S15: Split sample factor loadings for CFA with nine items (N = 466). Table S16: Full sample factor loadings for CFA with nine items (N = 934). Table S17: Model statistics for EFA models (N = 466). Table S18: Fit statistics for unconstrained and constrained model. [file CCH-52-e70199-s001.docx]

**PADTS Four Factor Model (Tables S5-S11)**

**Table S5: Split sample factor loadings for exploratory factor analysis with 20 Items (N=466)**

|  | **Factors** | | | | |  |
| --- | --- | --- | --- | --- | --- | --- |
|  | **1** | **2** | **3** | **4** | **5** | **Uniqueness** |
| AttitudesWellbeing_1R | 0.738 |  |  |  |  | 0.399 |
| AttitudesWellbeing_2R | 0.770 |  |  |  |  | 0.457 |
| AttitudesWellbeing_3Reverse |  | 0.634 |  |  |  | 0.594 |
| AttitudesWellbeing_4R | 0.752 |  |  |  |  | 0.403 |
| AttitudesWellbeing_5Reverse |  |  |  |  |  | 0.649 |
| AttitudesLearning_1Reverse |  | 0.726 |  |  |  | 0.440 |
| AttitudesLearning_2R | 0.466 |  |  |  |  | 0.430 |
| AttitudesLearning_3Reverse |  | 0.605 |  |  |  | 0.536 |
| AttitudesLearning_4R | 0.546 |  |  |  |  | 0.484 |
| AttitudesLearning_5Reverse |  | 0.588 |  |  |  | 0.502 |
| ParentConfidence_1r |  |  |  |  | 0.530 | 0.375 |
| ParentConfidence_2Reverse |  |  | 0.648 |  |  | 0.518 |
| ParentConfidence_3R |  |  |  |  | 0.492 | 0.500 |
| ParentConfidence_4Reverse |  |  | 0.731 |  |  | 0.401 |
| ParentConfidence_5Reverse |  |  | 0.752 |  |  | 0.392 |
| ParentAnxiety_1 |  |  |  | 0.428 | 0.530 | 0.469 |
| Parent_Anxiety_2_Reverse |  |  |  | 0.783 |  | 0.344 |
| ParentAnxiety_3 |  |  |  |  |  | 0.528 |
| Parent_Anxiety_4_Reverse |  |  |  | 0.688 |  | 0.472 |
| ParentAnxiety_5 |  |  |  |  |  | 0.709 |

**Table S6: Split sample factor loadings for exploratory factor analysis with 15 Items (N=466*)***

|  | **Factors** | | | |  |
| --- | --- | --- | --- | --- | --- |
| **Indicator** | **1** | **2** | **3** | **4** | **Uniqueness** |
| AttitudesWellbeing_1 | 0.668 |  |  |  | 0.430 |
| AttitudesWellbeing_2 | 0.434 |  |  |  | 0.572 |
| AttitudesWellbeing_3Reverse |  | 0.646 |  |  | 0.627 |
| AttitudesWellbeing_4 | 0.750 |  |  |  | 0.394 |
| AttitudesLearning_1Reverse |  | 0.758 |  |  | 0.432 |
| AttitudesWellbeing_2 | 0.766 |  |  |  | 0.479 |
| AttitudesLearning_3Reverse |  | 0.646 |  |  | 0.532 |
| AttitudesWellbeing_4 | 0.654 |  |  |  | 0.494 |
| AttitudesLearning_5Reverse |  | 0.618 |  |  | 0.500 |
| ParentConfidence_2Reverse |  |  | 0.649 |  | 0.536 |
| ParentConfidence_4Reverse |  |  | 0.763 |  | 0.398 |
| ParentConfidence_5Reverse |  |  | 0.781 |  | 0.397 |
| ParentAnxiety_1 |  |  |  | 0.618 | 0.569 |
| ParentAnxiety_3 |  |  |  | 0.568 | 0.510 |
| ParentAnxiety_5 |  |  |  | 0.627 | 0.661 |

**Table S7: Model Statistics for EFA Models (N=466)**

|  |  | **RMSEA 90% CI** | |  |  | **Model Test** | | |
| --- | --- | --- | --- | --- | --- | --- | --- | --- |
|  | **RMSEA** | **Lower** | **Upper** | **TLI** | **BIC** | **χ²** | **df** | **p** |
| **EFA – 20 items** | 0.0383 | 0.0281 | 0.0483 | 0.959 | -446 | 169 | 100 | < .001 |
| **EFA – 15 items** | 0.0590 | 0.0469 | 0.0714 | 0.924 | -180 | 134 | 51 | < .001 |

**Table S8: Split sample factor loadings for CFA with 15 Items (N=466)**

| **Indicator** | **Estimate** | **SE** | **Z** | **p** |
| --- | --- | --- | --- | --- |
| AttitudesWellbeing_1R | 0.918 | 0.0466 | 19.73 | < .001 |
| AttitudesWellbeing_2R | 0.746 | 0.0474 | 15.73 | < .001 |
| AttitudesWellbeing_4R | 0.953 | 0.0477 | 20.00 | < .001 |
| AttitudesWellbeing_2R | 0.787 | 0.0491 | 16.03 | < .001 |
| AttitudesWellbeing_4R | 0.871 | 0.0523 | 16.66 | < .001 |
| AttitudesWellbeing_3Reverse | 0.462 | 0.0378 | 12.21 | < .001 |
| AttitudesLearning_1Reverse | 0.541 | 0.0329 | 16.42 | < .001 |
| AttitudesLearning_3Reverse | 0.584 | 0.0362 | 16.13 | < .001 |
| AttitudesLearning_5Reverse | 0.530 | 0.0375 | 14.13 | < .001 |
| ParentConfidence_2Reverse | 0.719 | 0.0450 | 15.98 | < .001 |
| ParentConfidence_4Reverse | 0.793 | 0.0397 | 19.99 | < .001 |
| ParentConfidence_5Reverse | 0.834 | 0.0419 | 19.92 | < .001 |
| ParentAnxiety_1 | 0.742 | 0.0610 | 12.18 | < .001 |
| ParentAnxiety_3 | 0.744 | 0.0581 | 12.80 | < .001 |
| ParentAnxiety_5 | 0.615 | 0.0654 | 9.41 | < .001 |

**Table S9: Full sample factor loadings for CFA with 15 Items (N=932)**

| **Indicator** | **Estimate** | **SE** | **Z** | **p** |
| --- | --- | --- | --- | --- |
| AttitudesWellbeing_1R | 0.918 | 0.0466 | 19.73 | < .001 |
| AttitudesWellbeing_2R | 0.746 | 0.0474 | 15.73 | < .001 |
| AttitudesWellbeing_4R | 0.953 | 0.0477 | 20.00 | < .001 |
| AttitudesWellbeing_2R | 0.787 | 0.0491 | 16.03 | < .001 |
| AttitudesWellbeing_4R | 0.871 | 0.0523 | 16.66 | < .001 |
| AttitudesWellbeing_3Reverse | 0.462 | 0.0378 | 12.21 | < .001 |
| AttitudesLearning_1Reverse | 0.541 | 0.0329 | 16.42 | < .001 |
| AttitudesLearning_3Reverse | 0.584 | 0.0362 | 16.13 | < .001 |
| AttitudesLearning_5Reverse | 0.530 | 0.0375 | 14.13 | < .001 |
| ParentConfidence_2Reverse | 0.719 | 0.0450 | 15.98 | < .001 |
| ParentConfidence_4Reverse | 0.793 | 0.0397 | 19.99 | < .001 |
| ParentConfidence_5Reverse | 0.834 | 0.0419 | 19.92 | < .001 |
| ParentAnxiety_1 | 0.742 | 0.0610 | 12.18 | < .001 |
| ParentAnxiety_3 | 0.744 | 0.0581 | 12.80 | < .001 |
| ParentAnxiety_5 | 0.615 | 0.0654 | 9.41 | < .001 |

**Table S10: Model Statistics for CFA Models (N=932)**

| **CFA Model** |  |  |  |  | **RMSEA 90% CI** | |  |  | **Test for Exact Fit** | | |
| --- | --- | --- | --- | --- | --- | --- | --- | --- | --- | --- | --- |
|  | **CFI** | **TLI** | **SRMR** | **RMSEA** | **Lower** | **Upper** | **AIC** | **BIC** | **χ²** | **df** | **p** |
| **1 Factor (baseline)** | 0.439 | 0.373 | 0.143 | 0.156 | 0.152 | 0.160 | 52568 | 52859 | 4017 | 170 | < .001 |
| **4 Factor** | 0.625 | 0.565 | 0.125 | 0.130 | 0.125 | 0.134 | 51301 | 51620 | 2737 | 164 | < .001 |
| **Revised 4 Factor** | 0.948 | 0.935 | 0.0376 | 0.0565 | 0.0502 | 0.0629 | 35884 | 36131 | 334 | 84 | < .001 |

**Table S11: Fit Statistics for Unconstrained and Constrained Model**

|  | **Unconstrained Model** | | **Constrained Model** | |
| --- | --- | --- | --- | --- |
|  | CFI | RMSEA | CFI | RMSEA |
| Parent Gender | .939 | .062 | .933 | .059 |
| Parent Ethnicity | 944 | .059 | .938 | .056 |
| Child Age | .936 | 063 | .913 | .065 |
| UK Country | .924 | .069 | .916 | .064 |

**PADTS Three Factor Model (Tables S12-S18)**

**Table S12: Split sample factor loadings for exploratory factor analysis with 9 Items (N=466*)***

|  | **Factors** | | |  |
| --- | --- | --- | --- | --- |
|  | **1** | **2** | **3** | **Uniqueness** |
| AttitudesWellbeing_1R |  | 0.829 |  | 0.349 |
| AttitudesWellbeing_2R |  | 0.610 |  | 0.511 |
| AttitudesWellbeing_4R |  | 0.700 |  | 0.469 |
| ParentConfidence_2Reverse | 0.661 |  |  | 0.551 |
| ParentConfidence_4Reverse | 0.791 |  |  | 0.377 |
| ParentConfidence_5Reverse | 0.771 |  |  | 0.407 |
| ParentAnxiety_1 |  |  | 0.641 | 0.574 |
| ParentAnxiety_3 |  |  | 0.627 | 0.483 |
| ParentAnxiety_5 |  |  | 0.621 | 0.676 |

**Table S13: Model Statistics for EFA Models (N=466)**

|  |  | **RMSEA 90% CI** | |  |  | **Model Test** | | |
| --- | --- | --- | --- | --- | --- | --- | --- | --- |
|  | **RMSEA** | **Lower** | **Upper** | **TLI** | **BIC** | **χ²** | **df** | **p** |
| **EFA – 20 items** | 0.0383 | 0.0281 | 0.0483 | 0.959 | -446 | 169 | 100 | < .001 |
| **EFA – 9 items** | 0.0455 | 0.0165 | 0.0727 | 0.971 | -50.1 | 23.6 | 12 | 0.023 |

**Table S14: Model Statistics for EFA Models (N=466)**

| **CFA Model** |  |  |  |  | **RMSEA 90% CI** | |  |  | **Test for Exact Fit** | | |
| --- | --- | --- | --- | --- | --- | --- | --- | --- | --- | --- | --- |
|  | **CFI** | **TLI** | **SRMR** | **RMSEA** | **Lower** | **Upper** | **AIC** | **BIC** | **χ²** | **df** | **p** |
| **1 Factor (baseline)** | 0.439 | 0.373 | 0.143 | 0.156 | 0.152 | 0.160 | 52568 | 52859 | 4017 | 170 | < .001 |
| **4 Factor** | 0.625 | 0.565 | 0.125 | 0.130 | 0.125 | 0.134 | 51301 | 51620 | 2737 | 164 | < .001 |
| **3 Factor Full Sample** | 0.978 | 0.967 | 0.0289 | 0.0507 | 0.0388 | 0.0630 | 22890 | 23035 | 81.4 | 24 | < .001 |

**Table S15: Split sample factor loadings for CFA with 9 Items (N=466)**

| **Indicator** | **Estimate** | **SE** | **Z** | **p** |
| --- | --- | --- | --- | --- |
| AttitudesWellbeing_1R | 0.938 | 0.0478 | 19.61 | < .001 |
| AttitudesWellbeing_2R | 0.807 | 0.0472 | 17.09 | < .001 |
| AttitudesWellbeing_4R | 0.950 | 0.0496 | 19.15 | < .001 |
| ParentConfidence_2Reverse | 0.722 | 0.0451 | 16.01 | < .001 |
| ParentConfidence_4Reverse | 0.801 | 0.0401 | 19.94 | < .001 |
| ParentConfidence_5Reverse | 0.824 | 0.0425 | 19.38 | < .001 |
| ParentAnxiety_1 | 0.742 | 0.0614 | 12.08 | < .001 |
| ParentAnxiety_3 | 0.749 | 0.0584 | 12.82 | < .001 |
| ParentAnxiety_5 | 0.607 | 0.0659 | 9.20 | < .001 |

**Table S16: Full sample factor loadings for CFA with 9 Items (N=934)**

| **Indicator** | **Estimate** | **SE** | **Z** | **p** |
| --- | --- | --- | --- | --- |
| **Attitudes to Devices** |  |  |  |  |
| Digital devices are damaging to children’s mental health | 0.886 | 0.0337 | 26.3 | < .001 |
| Young children use digital technology too much, too early | 0.784 | 0.0336 | 23.3 | < .001 |
| Digital devices are damaging to children’s physical health | 0.890 | 0.0355 | 25.1 | < .001 |
| **Confidence** |  |  |  |  |
| I know where I can access support and advice around children’s digital usage* | 0.743 | 0.0343 | 21.7 | < .001 |
| I know how to keep my child safe when using digital technology* | 0.802 | 0.0307 | 26.2 | < .001 |
| I believe I have all the skills to support my child using digital devices* | 0.799 | 0.0316 | 25.3 | < .001 |
| **Anxiety** |  |  |  |  |
| I get anxious when my child is spending too long on digital devices. | 0.734 | 0.0415 | 17.7 | < .001 |
| I am concerned that excessive use of digital devices will negatively impact the amount of time my child spends socialising with other children and adults. | 0.786 | 0.0390 | 20.1 | < .001 |
| I worry about the inappropriate content that my child might access online | 0.617 | 0.0448 | 13.8 | < .001 |
| Items were scored on a 5-point Likert scale. Items marked with * are reverse coded. Higher total scores indicate more positive attitudes toward digital technology, greater parental confidence, and lower anxiety | | | | |

**Table S17: Model Statistics for EFA Models (N=466)**

| **CFA Model** |  |  |  |  | **RMSEA 90% CI** | |  |  | **Test for Exact Fit** | | |
| --- | --- | --- | --- | --- | --- | --- | --- | --- | --- | --- | --- |
|  | **CFI** | **TLI** | **SRMR** | **RMSEA** | **Lower** | **Upper** | **AIC** | **BIC** | **χ²** | **df** | **p** |
| **1 Factor (baseline)** | 0.439 | 0.373 | 0.143 | 0.156 | 0.152 | 0.160 | 52568 | 52859 | 4017 | 170 | < .001 |
| **4 Factor** | 0.625 | 0.565 | 0.125 | 0.130 | 0.125 | 0.134 | 51301 | 51620 | 2737 | 164 | < .001 |
| **3 Factor Full Sample** | 0.978 | 0.967 | 0.0289 | 0.0507 | 0.0388 | 0.0630 | 22890 | 23035 | 81.4 | 24 | < .001 |

**Table S18: Fit Statistics for Unconstrained and Constrained Model**

|  | **Unconstrained Model** | | **Constrained Model** | |
| --- | --- | --- | --- | --- |
|  | CFI | RMSEA | CFI | RMSEA |
| Parent Gender | .973 | .056 | .966 | .052 |
| Parent Ethnicity | 975 | .054 | .974 | .046 |
| Child Age | .974 | 056 | .957 | .057 |
| UK Country | .964 | .065 | .958 | .055 |
